# Role of the Serine-Rich Surface Glycoprotein Srr1 of *Streptococcus agalactiae* in the Pathogenesis of Infective Endocarditis

**DOI:** 10.1371/journal.pone.0064204

**Published:** 2013-05-23

**Authors:** Ho Seong Seo, Yan Q. Xiong, Paul M. Sullam

**Affiliations:** 1 Division of Infectious Diseases, Veterans Affairs Medical Center and the University of California San Francisco, San Francisco, California, United States of America; 2 Department of Medicine, Los Angeles Biomedical Research Institute at Harbor-UCLA Medical Center, Torrance, California, United States of America; 3 Geffen School of Medicine at UCLA, Los Angeles, California, United States of America; University of Florida, United States of America

## Abstract

The binding of bacteria to fibrinogen and platelets are important events in the pathogenesis of infective endocarditis. Srr1 is a serine-rich repeat glycoprotein of *Streptococcus agalactiae* that binds directly to the Aα chain of human fibrinogen. To assess the impact of Srr1 on the pathogenesis of endocarditis due to *S. agalactiae*, we first examined the binding of this organism to immobilized human platelets. Strains expressing Srr1 had significantly higher levels of binding to human platelets *in vitro*, as compared with isogenic Δ*srr1* mutants. In addition, platelet binding was inhibited by pretreatment with anti-fibrinogen IgG or purified Srr1 binding region. To assess the contribution of Srr1 to pathogenicity, we compared the relative virulence of *S. agalactiae* NCTC 10/84 strain and its Δ*srr1* mutant in a rat model of endocarditis, where animals were co-infected with the WT and the mutant strains at a 1∶1 ratio. At 72 h post-infection, bacterial densities (CFU/g) of the WT strain within vegetations, kidneys, and spleens were significantly higher, as compared with the Δ*srr1* mutant. These results indicate that Srr1 contributes to the pathogenesis of endocarditis due to *S. agalactiae*, at least in part through its role in fibrinogen-mediated platelet binding.

## Introduction

*Streptococcus agalactiae* (Group B streptococcus [GBS]) is a frequent cause of neonatal meningitis and sepsis. In recent years, however, GBS infections in nonpregnant adults are being increasingly reported. Individuals at greater risk for this disease include the elderly, immunosuppressed patients, and diabetics [1]–[3]. Although GBS is a relatively uncommon cause of endocarditis (accounting for 1–2% of culture-positive cases), endovascular infection due to this organism is associated with a high mortality rate (34–50%), especially in the setting of prosthetic valve infection [4]–[7]. Complications such as sepsis, valvular destruction, cardiac failure, and embolic phenomena are also frequent in this disease [8].

The pathogenesis of endocarditis is a complex process, involving multiple host-pathogen interactions. A central aspect of virulence in this disease is the ability of organisms to bind host components, such as fibrinogen, fibronectin, and platelets [9]–[13]. These binding events appear to be important both for the initial attachment of bacteria to the endovascular surface, and for the subsequent progression of infection. For several Gram-positive bacteria, binding to human platelets is mediated in part by an adhesin belonging to the serine-rich repeat (SRR) glycoprotein [14]–[16]. For example, strains of *Streptococcus gordonii* can bind platelets directly via the interaction of the SRR adhesins GspB or Hsa with a receptor (GPIb) on the platelet membrane [15]. The binding of *Staphylococcus aureus* to platelets is mediated in part by the SRR protein SraP, though the receptor for this adhesin remains unidentified [17]. In addition, *S. aureus* can attach to platelets via fibrinogen and fibrin, which act as a molecular bridge between the bacteria and platelet surface [18]–[20].

Two SRR glycoproteins (Srr1 and Srr2) have been identified thus far in *S. agalactiae* [21]. Expression of Srr1 has been shown to contribute to colonization and virulence in models of GBS bacteremia and meningitis infection [22]–[25]. In addition, we have recently demonstrated that Srr1 binds to human fibrinogen via its interaction with the Aα chain of the protein, and that loss of fibrinogen binding is associated with decreased attachment to brain microvascular endothelial cells *in vitro*, as well as attenuated virulence, in an experimental model of meningitis [26]. In view of the importance of fibrinogen binding for endovascular infection, we examined the impact of Srr1 on platelet binding *in vitro*, and its role in the pathogenesis of infective endocarditis.

## Materials and Methods

### Reagents

Purified human fibrinogen was obtained from Haematologic Technologies. Rat fibrinogen was purchased from Sigma-Aldrich. Rabbit anti-fibrinogen IgG was acquired from Innovative Research.

### Strains, plasmids, and growth conditions

The bacteria and plasmids used in this study are listed in Tables 1. *S. agalactiae* strains were grown in Todd-Hewitt broth (Difco) supplemented with 0.5% yeast extract (THY broth). All mutant strains grew at a comparable rate *in vitro* as compared with respective parental strains (data not shown). *Escherichia coli* strains DH5α, BL21 and BL21(DE3) were grown at 37°C under aeration in Luria Bertani broth (LB; Difco). Antibiotics were added to the media as required. All isolates were stored at −80°C until thawed just prior to use.

**Table 1:** Strains and plasmids.

Strains	Genotype or description^a^	Source
*Escherichia coli*		
DH5α	F^−^r^−^m^+^Ø80d*lacZ*ΔM15	Gibco BRL
BL21 (DE3)	expression host, inducible T7 RNA polymerase	Novagen
*Streptococcus agalactiae*		
COH31	serotype III, clinical isolate	[51]
PS954	COH31Δ*srr*1, Cm^R^	this study
NCTC 10/84	serotype V, clinical isolate	[52]
PS2645	NCTC 10/84Δ*srr*1, Cm^R^	[23]

Plasmids	Description^a^	Source
pDE123	streptococcal shuttle vector, Erm^R^	[26]
pDE123-*srr1*	vector for expression of Srr1, Erm^R^	[26]
pET22b(+)	expression vector, Amp^R^	Novagen
pET28_FLAG_	expression vector with FLAG-tag, Kan^R^	[27]
pET22-Srr1-BR	vector for expression of Srr1-BR, Amp^R^	[26]
pET28_FLAG_Srr1-BR	vector for expression of FLAG-tagged Srr1_303–641_	this study

a Cm^R^, chloramphenicol resistance; ErmR, erythromycin resistance; Amp^R^, ampicillin resistance; Kan^R^, kanamycin resistance.

### Cloning and expression of the Srr1 binding region (Srr1-BR)

Genomic DNA was isolated from GBS NCTC 10/84 using Wizard Genomic DNA purification kits (Promega), according to the manufacturer's instructions. PCR products were cloned into either pET28_FLAG_ or pET22(+) to express FLAG-tagged or His6-tagged versions of Srr1-BR (amino acids [AA] 303–641 of the SRR1). Proteins were purified by either Ni-NTA (Promega) or anti-FLAG M2 agarose affinity chromatography (Sigma-Aldrich).

### Binding of *S. agalactiae* to immobilized human platelets and rat fibrinogen

Overnight cultures of *S. agalactiae* were harvested by centrifugation, washed in PBS, and adjusted to a concentration of 10^6^ CFU/ml. Purified rat fibrinogen or washed, fixed human platelets were immobilized in 96-well microtiter plates as described previously (46). The plates were then treated with casein-based blocking solution (Roche) at 37°C for 1 h and washed three times with PBS. Purified recombinant Srr1-BR (200 µg/ml), anti-Srr1 IgG (100 µg/ml), or anti-fibrinogen IgG (100 µg/ml) were added to the wells for 30 min, followed by washing and the addition of 100 µl of the bacterial suspension. The plates were incubated at room temperature for 1 h, and the wells were washed three times with PBS to remove nonadherent organisms. The wells were treated with 100 µl of trypsin (2.5 mg/ml) for 10 min at 37°C to release the attached bacteria, and the number of bound bacteria was determined by plating serial dilutions of the recovered bacteria onto blood agar plates as previously described [27].

### Binding of recombinant Srr1-BR to human platelets

Fixed human platelets were immobilized in 96 well cell culture plates as described previously [28]. After treatment with a casein-based blocking reagent, the wells were incubated with _FLAG_Srr1-BR (0–4 µM) in PBS for 1 h at room temperature, followed by washing. Bound protein was detected by ELISA with anti-FLAG monoclonal antibody. For some studies, the wells were preincubated with His6-tagged Srr1-BR (0–50 µM) or anti-fibrinogen IgG (0–100 µg/ml) for 0.5 h at RT followed by washing, prior to adding _FLAG_Srr1-BR (1 µM).

### Rat model of infective endocarditis

The relative virulence of *S. agalactiae* NCTC 10/84 parental strain and its isogenic variant (NCTC 10/84 Δ*srr1*) was assessed in a competition model of IE in rats, as described previously [29], [30]. In brief, Sprague-Dawley female rats (250 to 300 g, Harlan Laboratory, Inc.) were first anesthetized with ketamine (35 mg/kg) and xylazine (10 mg/kg). A sterile polyethylene catheter was surgically placed across the aortic valve of each animal, such that the tip was positioned in the left ventricle, to induce the formation of sterile valvular vegetations (nonbacterial thrombotic endocarditis) [27], [29]. The catheters were left in place throughout the study. Three days post-catheterization, the animals were infected intravenously with an inoculum of approximately 5×10^5^ CFU containing a 1∶1 mixture of *S. agalactiae* NCTC 10/84 and its Δ*srr1* isogenic variant. At 72 hr post-infection, the rats were euthanized with thiopental (100 mg IP). Animals were included in the final analysis only if the catheters were correctly positioned across the aortic valve at the time of sacrifice, and if macroscopic vegetations were visible. At sacrifice, all cardiac vegetations, as well as kidneys and spleens, were harvested, weighed, homogenized in saline, serially diluted, and plated onto 8% sleep blood Todd Hewitt agar (with or without 2.5 µg/ml of chloramphenicol) for quantitative culture. The plates were incubated for 48 h at 37°C, and bacterial densities were expressed as the log_10_CFU per gram of tissue. Differences in means +/− SD were compared for statistical significance by the paired t-test. The data were also analyzed by calculating a “competition index,” which was defined as the ratio of the paired strains within tissues for each animal, normalized by the ratio of organisms in the inoculum [27], [29]. The mean of the log_10_ normalized ratios was tested against the hypothesized ‘no effect’ mean value of 0, using a paired t-test, with *P*≤0.05 as the threshold for statistical significance.

Animals were maintained in accordance with the American Association for Accreditation of Laboratory Animal Care (AAALAC) criteria. All animal studies were approved by the Animal Research Committee (IACUC) of the Los Angeles Biomedical Research Institute at Harbor-UCLA Medical Center.

### Statistical methods

Data expressed as means ± standard deviations were compared for statistical significance by the paired or unpaired *t* test, as indicated.

## Results

### Binding of GBS to human platelets is mediated by fibrinogen

**Figure 1:**
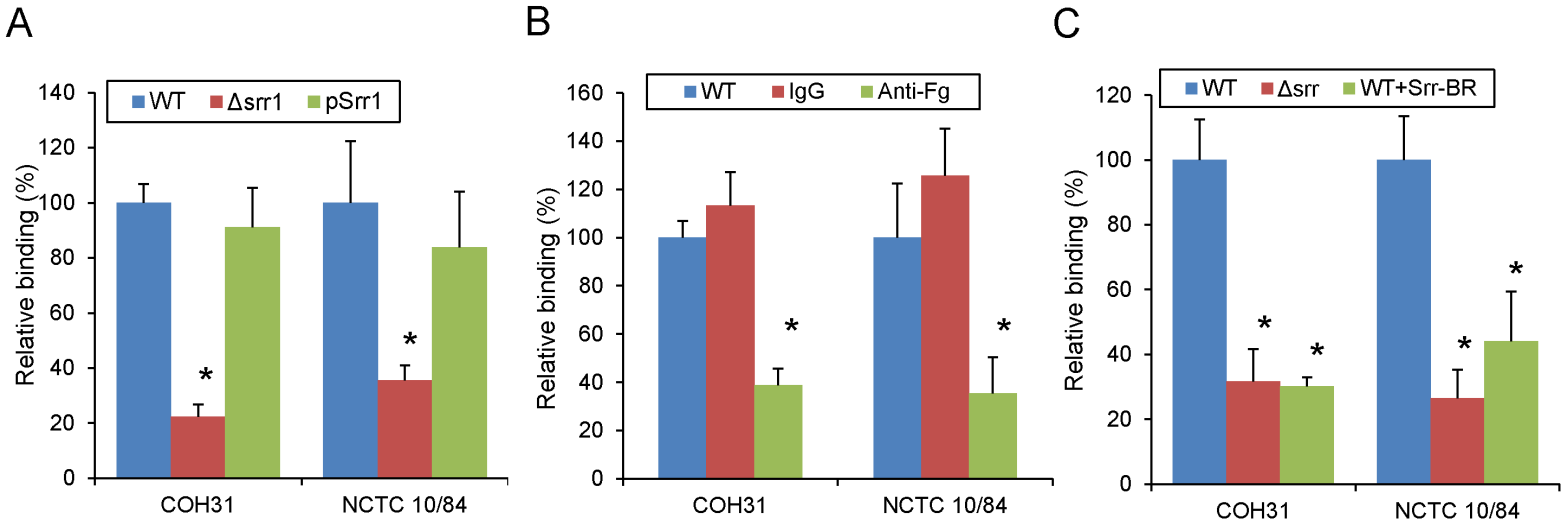
GBS binding to immobilized human platelets is mediated by glycoprotein Srr1. (A) Platelet binding by GBS strains COH31 and NCTC 10/84, their Δ*srr1* isogenic variants, and the mutant strains complemented *in trans* with *srr1* (pSrr1). (B) GBS binding to human platelets was inhibited by pretreating the monolayers with 100 µg/ml of anti-fibrinogen IgG (Anti-Fg). Normal IgG (IgG) served as a control. (C) Inhibition of binding by recombinant Srr1 binding region (Srr1-BR). Levels of binding were calculated as relative to the WT strains (mean ± SD). Values shown represent the means (± S.D.) of triplicate measurements. * = P<0.01.

To assess whether GBS binding to human platelets is mediated by Srr1, we compared two GBS strains (COH31 and NCTC 10/84) and their respective *srr1* deletion variants for adherence to these cells *in vitro* (Fig. 1A). Both strains bound platelets significantly above background levels, with 28.0±2.6% and 12.0±2.6% (mean ± SD) of the inoculum bound for COH31 and NCTC 10/84, respectively. Levels of binding by both *srr1* mutant strains were significantly lower than those of the parent strains, with a 79.2±3.4% reduction in platelet binding for COH31Δ*srr1* and a 71.4±4.2% reduction for NCTC 10/84Δ*srr1*. Complementation of the *srr1* mutation *in trans* restored binding by both mutant strains, thereby demonstrating that the loss of binding observed with *srr1* disruption was not due to polar or pleiotropic effects. In addition, GBS binding to human platelets was inhibited by rabbit anti-fibrinogen IgG, but not by normal rabbit IgG (Fig. 1B), with WT GBS binding levels reduced to those seen with the *srr1* deletion strains. We then examined the impact of preincubating the platelet monolayers with the recombinant binding domain of Srr1 (Srr1-BR). As shown in Fig. 1C, pretreating the immobilized platelets with recombinant Srr1-BR inhibited subsequent binding by both GBS strains. Since previous studies have shown that human platelets express membrane-associated fibrinogen [11], [31], [32], our results indicate that GBS binding to human platelets is mediated by the interaction of Srr1 with fibrinogen on the surface of these cells.

### Binding of Srr1-BR to immobilized platelets

**Figure 2:**
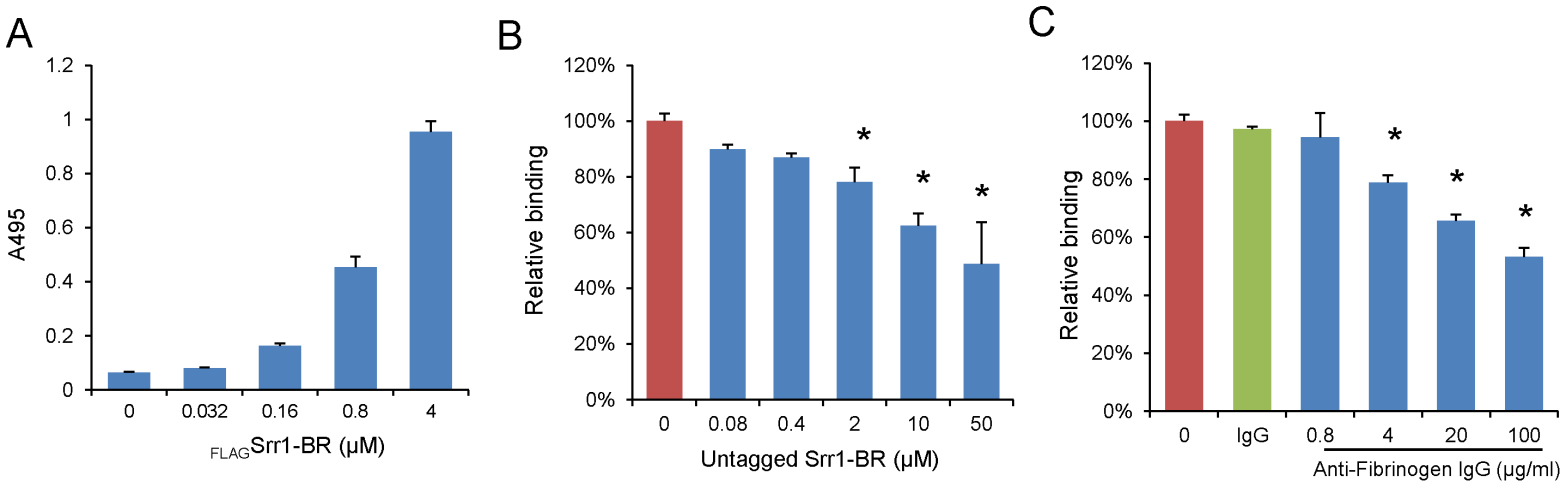
Recombinant Srr1-BR interacts with human platelets. (A) Binding of _FLAG_Srr1-BR protein to immobilized platelets. (B) Inhibition of _FLAG_Srr1-BR binding to platelets by His6 tagged Srr1-BR. Platelets were pretreated with the indicated concentrations of His6 tagged Srr1-BR. (C) Binding of _FLAG_Srr1-BR to immobilized platelets pretreated with anti-fibrinogen IgG or preimmune rabbit IgG. Values represent relative binding of _FLAG_Srr1-BR binding as compared with untreated platelets. Bars indicate the means (± S.D.). * = P<0.01.

To further assess the role of Srr1, we evaluated the binding of FLAG-tagged Srr1-BR (_FLAG_Srr1-BR) to immobilized human platelets. We found that _FLAG_Srr1-BR interacted with platelets in a concentration-dependent manner, when tested over a range of 0–4 µM (Fig. 2A). In addition, binding was significantly inhibited by preincubating the platelets with His6-tagged Srr1-BR, (Fig. 2B) or anti-fibrinogen IgG (Fig. 2C). These results demonstrate that Srr1-BR can bind platelets via its interaction with fibrinogen, and that this interaction is specific.

### Effect of Srr1 expression on streptococcal endocarditis

**Figure 3:**
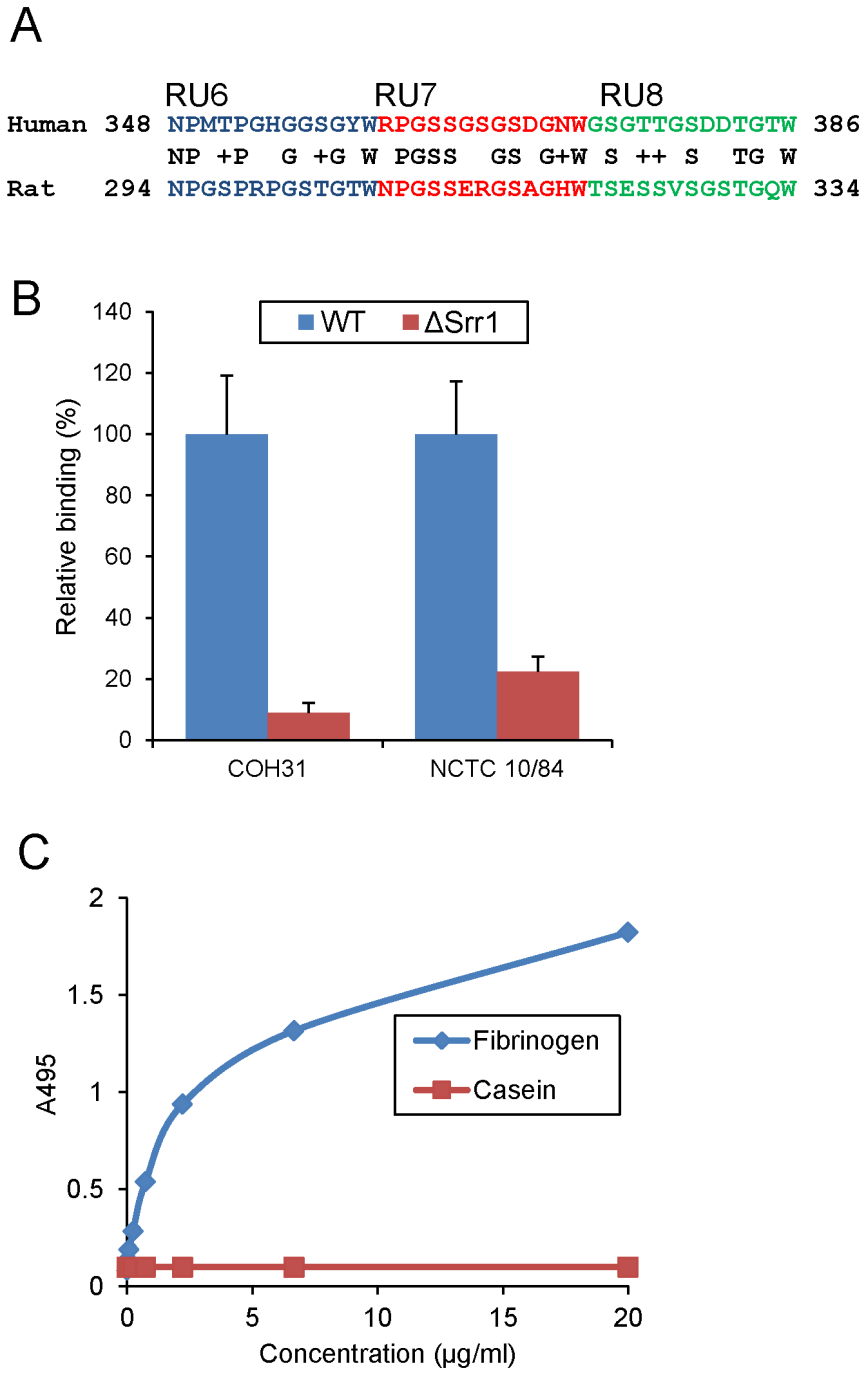
GBS binding to rat fibrinogen. (A) Alignment of Srr1 binding domain in the human fibrinogen Aα chain, with the homologous region of the rat protein; (B) Rat fibrinogen binding by wild type GBS and their isogenic variants (Δ*srr1*). (C) Rat fibrinogen binding by _FLAG_Srr1-BR protein over a range of concentrations. Casein served as a negative control.

Some fibrinogen binding proteins, such as ClfA of *S. aureus*, bind fibrinogen from only certain animal species [33]. With a view towards *in vivo* studies, we next sought to assess whether Srr1 had a similar impact on the interaction of GBS with rat fibrinogen. PSI-BLAST analysis indicated that the predicted binding region in rat fibrinogen is located at AA294–334 of the Aα chain, which has 49% identity with the Srr1 binding site on human fibrinogen (Fig. 3A). When tested *in vitro*, binding of the isogenic mutants to rat fibrinogen was found to be significantly lower than that of wild type strains COH31 and NCTC 10/84 (Fig. 3B). In addition, _FLAG_Srr1-BR was bound to immobilized rat fibrinogen in a concentration-dependent manner, as was seen previously with human fibrinogen (Fig. 3C) [26].

**Figure 4:**
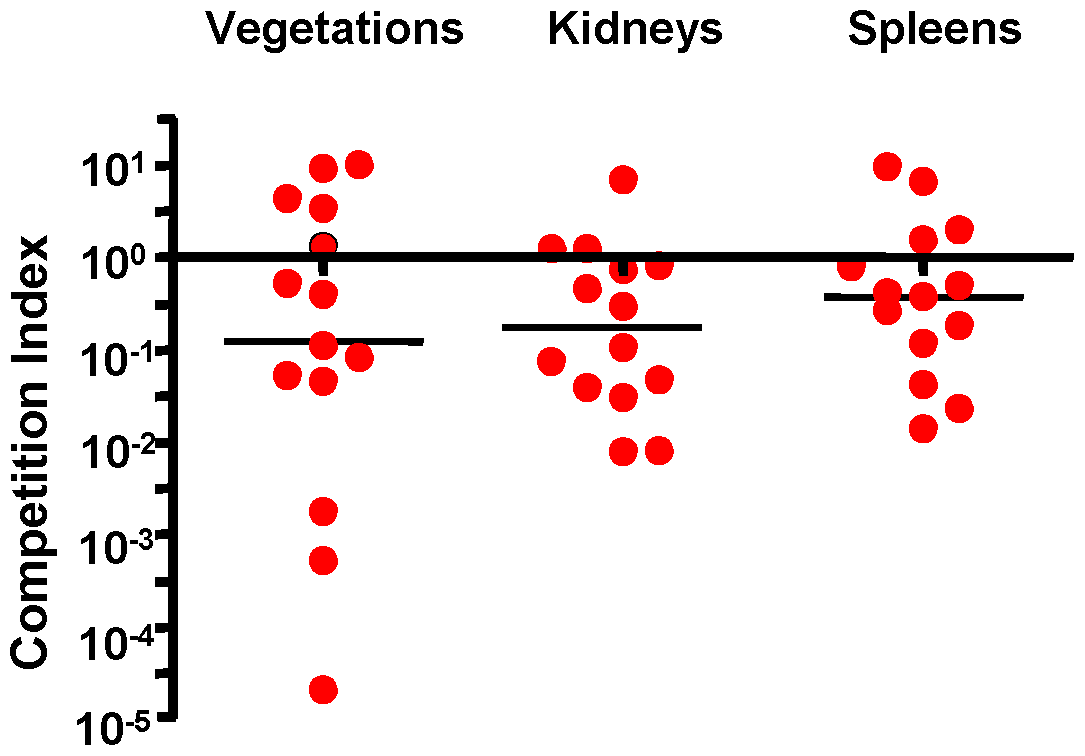
Competitive index (CI) analysis of WT and Δ*srr1* mutant obtained in the rat model of endocarditis. Competition index (CI) was calculated as the ratio of the WT to the Δ*srr1* mutant in each tissue, normalized for the ratio of strains within the inoculum. Circles represent data from individual animals. A CI above 10^0^ (dashed line) indicates a competitive disadvantage of Δ*srr1* compared with WT. Horizontal black bars indicates means of CIs.

To examine the impact of Srr1 expression on the pathogenesis of endocarditis, we compared the relative virulence of GBS NCTC 10/84 with its isogenic mutant (Δ*srr1*), as measured by a rat co-infection model of this disease. Animals (n = 14) had significantly lower densities of the mutant strain (mean log_10_ CFU/g ± SD = 7.46±1.63) within vegetations as compared with the parent strain (8.62±1.25). Levels of the mutant strain were also significantly reduced within kidneys and spleens (Table 2). We then re-analyzed these data by comparing the ratio of the isogenic strains within tissues, with the CFU of each strain normalized to the number of CFU within the inoculum (competition index) (Fig. 4). When assessed by this approach, the levels of the *srr1* mutant (Δ*srr1*) remained significantly reduced in all tissues, as compared with WT. Thus, Srr1 appears to be a significant virulence determinant for the pathogenesis of endocarditis due to GBS.

**Table 2:** Impact of Srr1 on virulence in an animal model of endocarditis.

	Vegetations		Kidneys		Spleens	
	Mean ± S.D. (log_10_CFU/g)	*p* value	Mean ± S.D. (log_10_CFU/g)	*p* value	Mean ± S.D. (log_10_CFU/g)	*p* value
**NCTC 10/84**	8.62±1.25	0.014	6.85±0.73	0.006	6.51±0.89	0.029
**Δ** ***srr1***	7.46±1.63		6.08±0.86		5.96±0.80	

Infective endocarditis was induced in rats, using an inoculum of 5×10^5^ CFU containing GBS NCTC10/84 and its isogenic Δ*srr1* mutant, at a 1∶1 ratio. Animals were sacrified 72 h post-infection, and log_10_ CFU/g of tissue for each strain was determined by plating onto selective media.

## Discussion

A number of bacterial surface structures have been shown to mediate binding to fibrinogen, such as ClfA, ClfB, FnbA and Efb of *S. aureus*, and the Fss proteins of *Enterococcus faecalis* [33]–[38]. We recently identified Srr1 of GBS as a fibrinogen-binding protein that was important for bacterial attachment to microvascular endothelial cells and CNS invasion [26]. Although the binding region of Srr1 has limited homology to other adhesins, analysis of its predicted secondary structure indicated that the conformation of this domain would resemble the binding region of ClfB. As has been shown for several other adhesins, the binding pocket of ClfB is formed by two Ig folds that engage the Aα chain of fibrinogen via a “dock, lock, and latch” mechanism [35], [39]. Srr1 appears to interact with fibrinogen Aα by a similar mechanism, since deletion of the predicted latch region abrogates fibrinogen binding by the protein, and markedly reduces virulence in an animal model of meningitis [26].

Our results indicate that Srr1-mediated binding to fibrinogen also contributes to the pathogenesis of infective endocarditis. Reduced fibrinogen binding *in vitro* was associated with decreased virulence, as measured by our co-infection (competition) model of endovascular infection. In particular, densities (CFU/g) of an Srr1 deletion mutant were significantly lower, as compared with its parent strain, both within vegetations and in kidneys and spleens. Of note, the mutant strain was not entirely avirulent, as it still produced disease in the infected animals. This indicates that GBS expresses other factors that contribute to its virulence, and is consistent with other studies on the role of microbial binding in endocarditis, where mutation or deletion of a single adhesin produces only a partial reduction in pathogenicity [17], [29], [30]. GBS are known to express other fibrinogen binding proteins (FbsA and FbsB), which may have contributed to the residual virulence of our Δ*srr-1* mutant strain [37], [38]. Moreover it is likely that GBS express additional surface components that can mediate binding to cardiac valves, or enhance virulence by other mechanisms.

Binding to fibrinogen may be important for a number of events in the pathogenesis of endovascular infections [10], [40], [41]. First, bacterial attachment to the endocardium generally requires prior alteration of the valve surface, such that it is covered with a matrix of platelets and host proteins, including fibrinogen [20], [42]–[45]. Studies with *S. aureus* have shown that fibrinogen immobilized on the valve surface is likely to contribute to the attachment of circulating bacteria, thereby initiating infection [46]–[50]. Our current results indicate that fibrinogen may have a similar role for GBS. In addition, fibrinogen in plasma could also serve to crosslink GBS to platelets that have aggregated at sites of valve injury. The subsequent progression of endovascular infection may also be enhanced by GBS binding to fibrinogen. Bacteria proliferating on the valve surface bacteria are thought to induce the further deposition of fibrinogen onto the infected valve, which in turn, is likely to trigger platelet attachment and aggregation. These processes, in combination with bacterial growth, result in the production of vegetations [9], [10]. In view of our *in vitro* studies, where Srr-1 enhanced the binding of bacteria to both fibrinogen and platelets, is possible that Srr1-fibrinogen binding may be one mechanism for the continued recruitment of platelets *in vivo* to the infected endocardium, thereby stimulating disease progression.

A longstanding therapeutic goal has been to develop agents that block bacterial binding to host tissues, thereby preventing or attenuating subsequent infection. Fibrinogen binding is an appealing target for disruption, in view of the importance of this interaction for the pathogenesis of infective endocarditis. Although inhibitory agents could target specific adhesins individually, such as Srr1, an alternative strategy might be to develop drugs that interfere with a larger number of “dock, lock, and latch” adhesins. Although the binding clefts of these adhesins vary in terms of primary amino acid sequence, it may still be possible to generate agents that block binding, either by preventing docking, or by inhibiting the latching process. If successful, this approach would yield an inhibitor that could be used for a variety of pathogens.
